# Multi-center transferability of a breath-hold T2 technique for myocardial iron assessment

**DOI:** 10.1186/1532-429X-10-11

**Published:** 2008-02-21

**Authors:** Taigang He, Paul Kirk, David N Firmin, Wynnie M Lam, Winnie CW Chu, Wing-Yan Au, Godfrey CF Chan, Ru San Tan, Ivy Ng, Selen Biceroglu, Yesim Aydinok, Mark A Fogel, Alan R Cohen, Dudley J Pennell

**Affiliations:** 1 National Heart and Lung Institute, Imperial College London, UK; 2 Cardiovascular Magnetic Resonance Unit, Royal Brompton Hospital, London, UK; 3 Department of Diagnostic Radiology & Organ Imaging, Prince of Wales Hospital, the Chinese University of Hong Kong, China; 4 Department of Medicine, Queen Mary Hospital, University of Hong Kong, China; 5 Department of Pediatrics and Adolescent Medicine, Queen Mary Hospital, University of Hong Kong, China; 6 National Heart Center, Singapore; 7 KK Women's and Children's Hospital, Singapore; 8 Department of Pediatric Hematology & Radiology, Ege University Hospital, Izmir, Turkey; 9 Children's Hospital of Philadelphia, USA

## Abstract

**Background:**

Cardiac iron overload is the leading cause of death in thalassemia major and is usually assessed using myocardial T2* measurements. Recently a cardiovascular magnetic resonance (CMR) breath-hold T2 sequence has been developed as a possible alternative. This cardiac T2 technique has good interstudy reproducibility, but its transferability to different centres has not yet been investigated.

**Methods and Results:**

The breath-hold black blood spin echo T2 sequence was installed and validated on 1.5T Siemens MR scanners at 4 different centres across the world. Using this sequence, 5–10 thalassemia patients from each centre were scanned twice locally within a week for local interstudy reproducibility (n = 34) and all were rescanned within one month at the standardization centre in London (intersite reproducibility). The local interstudy reproducibility (coefficient of variance) and mean difference were 4.4% and -0.06 ms. The intersite reproducibility and mean difference between scanners were 5.2% and -0.07 ms.

**Conclusion:**

The breath-hold myocardial T2 technique is transferable between Siemens scanners with good intersite and local interstudy reproducibility. This technique may have value in the diagnosis and management of patients with iron overload conditions such as thalassemia.

## Introduction

The development of transfusion related iron overload in the tissue of myocardium can result in cardiomyopathy [1,2], and heart failure remains the leading cause of death in beta-thalassemia major (TM) [3,4]. Myocardial iron measurement is important for assessing the risk of cardiac complications [5,6], because of the frequency of myocardial siderosis in thalassemia major [2], and the benefit of tailoring appropriate iron-chelating treatment [7-10].

Cardiovascular magnetic resonance (CMR) provides a non-invasive means of measuring the amount of tissue iron. With CMR, the iron deposition results in shortening of proton relaxation times and both T2 and T2* measurements have been exploited to assess iron overload [7,11]. Myocardial T2* is fast and simple to implement and has demonstrated good reproducibility in assessing tissue iron content [12-14]. A similar T2 sequence, however, is technically more difficult, and early attempts at implementation were unsatisfactory for myocardial iron measurements due to problems associated with hardware constraints, flow, cardiac motion, and noise [15,16]. However, T2 is known to vary with myocardial iron [17,18], and there may be potential advantages of T2 over T2* for the avoidance of problems caused by factors such as shimming and local susceptibility. There is also interest in comparing the myocardial T2* with T2 to discover if additional useful clinical information can be gleaned as to the chemical state of the stored iron. For this purpose, a breath-hold T2 technique has been developed and recently reported [19]. This T2 technique has demonstrated good interstudy reproducibility in London. However, in order to be applicable in healthcare on a wider scale, its transferability between scanners of different centers must be established, but no data on this challenging issue is available to date. We now describe the results of the transfer of this technique to different scanners at 4 different centers in different countries.

## Methods

### MR protocols and study population

The standard center in London used a 1.5T Siemens Sonata scanner. Four other international centers using 1.5T MR scanners were involved in this study. Scanner details were as follows: Center 1, Siemens Sonata (Hong Kong, China); Center 2, Siemens Avanto (Philadelphia, USA); Center 3 Siemens Avanto (Singapore); Center 4, Siemens Symphony Quantum (Izmir, Turkey). The breath-hold black blood T2 sequence developed at London center was installed on each local scanner. The pulse sequence details and methods of T2 calculation have been previously described [19]. In brief, all centers used a four-element cardiac phased array coil to image a single 10 mm mid-ventricular short axis slice at 12 echoes times (ranging from 4.8 ms to 163.2 ms, increment 14.4 ms) with ECG gating. Double inversion recovery pulses were applied to suppress the blood signal and data was acquired every other cardiac cycle. For T2 measurement, a region of interest (ROI) was chosen in the left ventricular septum individually. The mean signal intensity of ROI was measured for each of the images, and the data were plotted against the TEs to form a decay curve. The mono-exponential decay model and the nonlinear curve fitting algorithm were used to fit the curve to obtain T2 measurement. The sequence parameters were kept identical in all centers although small variations would not be expected to affect the T2 measurement.

A total of 34 (mean age 32 ± 9 years) TM patients (10 from Hong Kong, 10 from Singapore, 9 from Philadelphia, 5 from Izmir) were studied. For local interstudy reproducibility, these patients were scanned twice at their local centers within one week. All patients were subsequently rescanned at the standardization center at London (intersite reproducibility) within four weeks of their original scans. These patients had been regularly transfused since early childhood or since the introduction of deferoxamine, and were receiving regular iron chelation. Ethical approval was granted at the standard center and all local centers involved. All patients gave written informed consent.

### Statistics

To quantify both interstudy reproducibility and intersite transferability, the coefficient of variation was calculated (CoV), defined as standard deviation of the differences between the two separate measurements, divided by their mean and expressed as a percentage. Mean differences in T2 between scans are also quoted as a measure of bias. Summary data were expressed with 95% confidence intervals and displayed graphically using scatter plots with line of identity and Bland-Altman plots. Group data were compared using Friedman's test. A *p *value of < 0.05 was considered statistically significant.

## Results

Myocardial T2 measurements (two at each local site and one in London) were 46.4 ± 26.3 ms, 46.4 ± 26.1 ms and 46.3 ± 26.4 ms respectively. Friedman's test showed no significant difference (p = 0.87, n = 34) between these three T2 measurements.

### Local interstudy reproducibility

**Figure 1:**
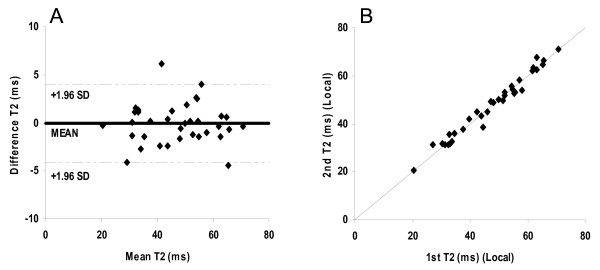
**Assessment of interstudy reproducibility at the centers (n = 34).** A) Bland-Altman plot of the myocardial T2 values obtained locally twice within a week, with the 95% confidence intervals shown as a dotted line. B) Scatter plot of the myocardial T2 values obtained locally twice within a week. The diagonal line shows the line of identity.

The mean difference was -0.06 ms and the CoV for local interstudy reproducibility was found to be 4.4%. These values are consistent with those of our previous published data [19]. There was no bias between the two measurements (Figure 1).

### Intersite reproducibility

**Figure 2:**
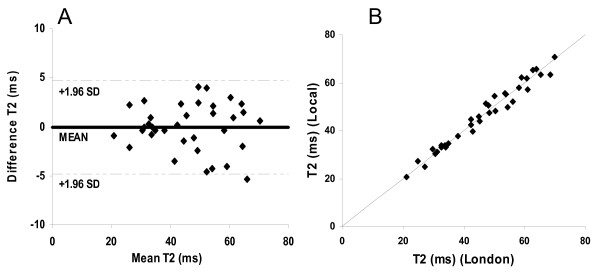
**Assessment of intersite reproducibility between local centers and the standard center in London (n = 34).** A) Bland-Altman plot of the myocardial T2 values obtained locally and at London within a month, with the 95% confidence intervals shown as a dotted line. B) Scatter plot of the myocardial T2 values obtained locally and at London within a month. The diagonal line shows the line of identity.

The mean difference was -0.07 ms and the CoV for intersite reproducibility was found to be 5.2%. These values were similar to those of local interstudy reproducibility mentioned above. This demonstrates good agreement between the standard center scanner and the local scanners at each center and no bias was found (Figure 2).

## Discussion

This study shows that the myocardial T2 values using the recently developed breath-hold T2 sequence agree very well between 1.5 Tesla scanners in different locations around the world. This demonstration of transferability suggests that this technique could be further disseminated worldwide, should its clinical applicability be demonstrated in iron overload conditions. Although the iron deposition affects both T2 and T2* relaxation times, T2 is not affected by extrinsic magnetic field inhomogeneity. Therefore, T2 in principle might provide more accurate measurements. Nevertheless, T2* has substantial clinical validation both in heart and liver suggesting that the potential limitations are more theoretical than real.[19] One potential advantage of T2 might be to perform robust multi-segmental analysis to explore the regional iron distribution, because T2* is currently limited to the septum to avoid susceptibility effects from the anterior and posterior cardiac veins and lungs. This may in due course improve our understanding of how other factors affect T2* across the myocardium. It is possible that different chemical forms of iron in the tissue could have differential effects between T2 and T2*, and measurement of both parameters might be useful if chelators access different forms of iron at different rates. From our studies of both T2* and T2, it is likely that both parameters can assess the tissue iron accurately in the heart using state-of-the-art sequences, and combined analysis may provide more information for patient management. Further research is needed to determine this. One limitation of this study was that only Siemens scanners were involved, and results from other scanners would be useful. There is no substantial barrier to this once the sequence has been adapted by other manufacturers.

To conclude, this study has demonstrated the good intersite reproducibility of the breath-hold T2 technique between 1.5T Siemens MR scanners at different centers across different countries. With widespread application, this T2 technique might yield new insights into the assessment of myocardial iron and may have clinical application possibly in conjunction with T2*.

## Competing interests

The author(s) declare that they have no competing interests.
